# Long non-coding RNA *THOR* promotes ovarian Cancer cells progression via IL-6/STAT3 pathway

**DOI:** 10.1186/s13048-020-00672-1

**Published:** 2020-06-17

**Authors:** Jing Ge, Tao Han, Lili Shan, Jing Na, Ya Li, Jun Wang

**Affiliations:** 1 Department of Gynecology and Obstetrics, Second Affiliated Hospital of Dalian Medical University, Dalian, 116023 Liaoning Province China; 2 Department of Gynecology and Obstetrics, General Hospital of Northern Theater Command, Shenyang, 110016 Liaoning Province China; 3 Department of Oncology, General Hospital of Northern Theater Command, Shenyang, 110016 Liaoning Province China

**Keywords:** Ovarian cancer, THOR, IL-6/STAT3, Progression, Prognosis

## Abstract

**Background:**

Ovarian cancer (OC) is one of the most common malignant tumors in the world. The prognosis of OC remains poor due to the advanced stage and distant metastasis at the time of diagnosis. Recently, a novel lncRNA, THOR (testis-associated highly conserved oncogenic long non-coding RNA), was characterized in human cancers and shown to exhibit an oncogenic role. However, the role of THOR in OC remains unclear.

**Methods:**

RT-PCR and western blot analysis were used to detect the expression of THOR, p-STAT3 and IL-6. The impact of THOR on OC proliferation, metastasis and self-renewal was investigated in vitro and in vivo. The prognostic value of THOR was determined in OC patient cohorts.

**Results:**

In this study, our results find that THOR is markedly upregulated in human OC tissues and predicts the poor prognosis of OC patients. Functional studies have revealed that knockdown of THOR inhibits the growth, metastasis and self-renewal of OC cells. Mechanistically, THOR drives OC cell progression via the IL-6/STAT3 signaling. Moreover, the specific STAT3 inhibitor S3I-201 or IL-6R inhibitor tocilizumab diminish the discrepancy in the growth, metastatic and self-renewal capacity between THOR-silenced OC cells and control cells, which further confirm that IL-6/STAT3 is required in THOR-driven OC cells progression.

**Conclusion:**

Our findings reveal that THOR could promote OC cells growth, metastasis and self-renewal by activating IL-6/STAT3 signaling and may be a good predictive factor and therapeutic target.

## Background

Ovarian cancer (OC) was one of the most common deadly malignancies in the world [1, 2]. More than 70% OC patients were diagnosed at advanced stage and lost their best operative chance [3]. Increasing evidence showed that tumor suppressor genes or proto-oncogenes, chromosomal and microsatellite instability, and epigenetic modifications have important roles in OC initiation and progression [4]. While the molecular and cellular mechanisms underlying the development of OC remains unclear. The lack of complete understanding of the molecular pathogenesis of OC has prevented the design of mechanism-based therapeutic recipes. Therefore, elucidation of the underlying mechanisms of initiation and progression of OC and identification of new therapeutic targets for OC are urgently needed.

Long non-coding RNAs (lncRNAs) are a heterogeneous class of transcripts with a length of more than 200 bases without protein-coding potential [5, 6]. Accumulating evidence has indicated that lncRNAs can affect disparate cellular functions and participate in diverse physiological and pathological processes [7]. The aberrant expression of lncRNAs has been demonstrated in multiple malignancies, including liver cancer, breast cancer, lung cancer and colorectal cancer [8], providing new insights into the pathogenesis of cancer [9]. The lncRNA THOR is reported as an oncogene, and transgenic THOR knockout produced fertilization defects in zebrafish and conferred resistance to melanoma initiation. The researchers also discovered a conserved interaction of THOR with IGF2BP1 and showed that THOR contributes to the mRNA stabilization activities of IGF2BP1 [10]. Previous studies also reported that THOR was upregulated in liver cancer and promoted HCC cells progression [11]. However, the role of THOR in OC initiation and progression remains unknown.

In the present study, we for find that THOR is highly expressed in OC patients and associates with poor prognosis of OC patients. Next, by using loss-of-function analysis in OC cells, we demonstrate that THOR promotes the proliferation, metastasis and self-renewal of OC cells. Further mechanistic analysis reveals that the IL-6/STAT3 pathway is activating by THOR in OC cells. Moreover, the special STAT3 inhibitor S3I-201 or IL-6R inhibitor tocilizumab abolish the discrepancy in growth, metastatic and self-renewal capacity between THOR-silenced OC cells and control cells. Collectively, the data shows that THOR promotes the progression of OC cells via the IL-6/STAT3 cascades and could be a good predictive factor and a potential therapeutic target of OC patients.

## Methods

### Patients and tissue samples

The OC patients’ tumor tissues and paired non-tumor tissues were obtained from patients who underwent curative surgery at General Hospital of Northern Theater Command. The specimens were frozen in liquid nitrogen immediately and then stored at − 80 °C. Overall survival (OS) was defined as the time interval between the date of surgery to the date of death or the last follow-up. Cumulative recurrence was defined as the time interval between the dates of surgery to the date of diagnosis of recurrence. A total of 90 tissue samples were also used for clinical prognosis analysis. Patient informed consent was also obtained and the procedure of human sample collection was approved by the Ethic Committee of General Hospital of Northern Theater Command.

### Cell lines and culture

SKOV3, A2780, OVCA429, 3AO, PEO-1, HO8910 and HGSOC cells were purchased from Chinese Academy of Sciences, Shanghai, China. The ovarian cancer cells were cultured in RPMI-1640 medium supplemented with 10% fetal calf serum (FCS; Invitrogen, Carlsbad, CA, USA) at 37 °C in a 5% CO_2_ incubator. The cultured cells were trypsinized with 0.5% trypsin and moved to a new six-well plate three times a week.

HO8910 or HGSOC cells were seeded into a six-well plate until they reached 60–70% confluence. The cells were infected with shTHOR lenti-virus and control virus as described previously [12]. The sequence of shTHOR is as follows: 5′- GGUGAACACAAUCGAGCAATT-3′. The shRNA lenti-virus was purchased from Shanghai GenePharma (Shanghai, China).

HO8910 shTHOR or HGSOC shTHOR cells and their control cells were treated with S3I-201 (100 nM) or not and then subjected to CCK8, Transwell (24 h), invasion assays (48 h) and spheroids formation (7 days).

HO8910 shTHOR or HGSOC shTHOR cells and their control cells were treated with tocilizumab (20 μg/mL) or not and then subjected to CCK8, invasion assays (48 h) and spheroids formation (7 days).

### Cell proliferation assays

HO8910 shTHOR or HGSOC shTHOR cells and their control cells were seeded in 96-well plates (3 × 10^3^ cells/well). ATP activity was measured using CCK8 assays at the indicated time points. The procedure was as follows: The cell suspension (100 μl/well) was inoculated in a 96-well plate, and the plate was pre-incubated in a humidified incubator at 37 °C for 1 h. This was followed by the addition of 10 μl of the CCK-8 solution to each well of the plate, and incubation of the plate for 1 h in the incubator. Finally, the absorbance was measured at 450 nm using a microplate reader (Synergy H1; BioTek Instruments, Inc., Winooski, VT, USA).

### Colony formation assay

For colony formation assays, the HO8910 shTHOR or HGSOC shTHOR cells and their control cells were seeded in 12-well plates (3000 cells/well). The cells were incubated for 7 days and then fixed with 10% neutral formalin for more than 4 h. The cells were dyed with crystal violet (Beyotime, Haimen, China). The cells were then photographed under a microscope (Olympus, Tokyo, Japan).

### EdU immunofluorescence staining

For cell EdU immunofluorescence staining, HO8910 shTHOR or HGSOC shTHOR cells and their control cells were seeded into 96-well plates and examined using the EdU kit (RiboBio, Guangzhou, China). The results were quantified with a Zeiss Axiophot photomicroscope (Carl Zeiss, Jena, Germany) and Image-Pro Plus 6.0 software.

### Cell migration assays

For cell migration experiments, 2 × 10^5^ OC cells were seeded into the upper chamber of a 24-well polycarbonate transwell in serum-free DMEM. The lower chamber was supplemented with DMEM containing 20% FBS as chemoattractant. The cells were incubated for 24 h, and the chamber was fixed with 10% neutral formalin for more than 4 h. The cells were dyed with crystal violet (Beyotime). Then the cells were counted under a microscope (Olympus), and the cell number is expressed as the average number of the cells in 5 fields.

### Cell invasion assays

For cell invasion experiments, 2 × 10^5^ OC cells were seeded into the upper chamber of a Matrigel-coated Boyden chamber in serum-free DMEM. The lower chamber was supplemented with DMEM containing 20% FBS as a chemoattractant. The cells were incubated for 48 h, and the chamber was fixed with 10% neutral formalin for more than 4 h. The cells were dyed with crystal violet (Beyotime). Then the cells were counted under a microscope (Olympus), and the cell number is expressed as the average number of cells in each field.

### Flow-cytometry analysis

For CD133^+^ cells sorting, HO8910 and HGSOC cells were incubated with the primary anti–CD133 (Cat. no. 372806, Biolegend, Inc., San Diego, CA) for 30 min at room temperature. The cells were then subjected to flow cytometry using a MoFlo XDP cell sorter from Beckman Coulter (Indianapolis, IN, USA) according to the manufacturer’s instructions. The sorted cells from three independent experiments were subjected to Real-time PCR assay.

### Spheroid formation assay

HO8910 shTHOR or HGSOC shTHOR cells and their control cells were cultured in a 6-well or 96-well ultra-low attachment culture plate for 1 week, and the total number of spheres was counted under the microscope.

### Real-time PCR

The total RNA from OC cells or the OC patient tissues was extracted by using TRIzol reagent (Invitrogen, 15,596–018). Total cDNAs were synthesized by a ThermoScript™ RT-PCR system (Invitrogen, 11,146–057). The total mRNA amount present in the cells was measured by RT-PCR using the ABI PRISM 7300 sequence detector (Applied Biosystems). The THOR primer sequences were as follows: forward: 5′-ACAATCGAGCAAGGCAGTGA-3′, reverse: 5′-TGGCCAAGACCTGCTGTTAG-3′. β-actin was used as reference for relative expression calculation and its primer sequences were as follows: forward: 5′-GGCCCAGAATGCAGTTCGCCTT-3′, reverse: 5′-AATGGCACCCTGCTCACGCA-3′. The PCR cycling conditions were as follows: 94 °C degeneration for 10 min; 94 °C modification for 30 s, 60 °C annealing for 30 s, 72 °C extension for 40 s, total of 40 cycles, 72 °C terminal extension for 10 min.

### Western blotting assays

The OC cells or OC patients’ tissues were lysed with cell lysis buffer (Beyotime) followed by supersonic splitting as described previous [13]. The total protein was quantified using a BCA Protein Quantification kit. A total of 25 μg of protein was subjected to sodium dodecyl sulfate polyacrylamide gel electrophoresis and then transferred onto nitrocellulose membranes. The membranes were blocked with 10% non-fat milk and incubated with primary antibodies overnight. The protein band, specifically bound to the primary antibody, was detected using an IRDye 800CW-conjugated secondary antibody and the LI-COR imaging system (LI-COR Biosciences, Lincoln, NE, USA). The primary antibodies were p-AKT (1:1000; #4060, Cell Signaling Technology), p-STAT3 (1:1000; #9145, Cell Signaling Technology), p-MEK (1:1000; #9127, Cell Signaling Technology), p-SMAD3 (1:1000; #9520, Cell Signaling Technology), IL-6 (1:1000; #12912, Cell Signaling Technology) and GAPDH (1:5000; #5174, Cell Signaling Technology).

### In vivo animal models

The NOD-SCID mice were purchased from Slake Company, Shanghai academy of sciences. All mouse experiments were performed according to the guidelines of the animal care and use committees at Shanghai Baoshan District Hospital of Integrated Chinese and Western medicine.

For xenograft formation assay, HO8910 shTHOR or its control cells (2X10^6^) were injected subcutaneously into nude mice. Mice were sacrificed 6 weeks post inoculation and tumors were collected and examined.

For pulmonary metastasis assay, HO8910 shTHOR or its control cells (2 X10^6^) were injected into the tail vein of nude mice. The mice were sacrificed 12 weeks post inoculation and consecutive sections of the whole lung were subjected to hematoxylin and eosin (H&E) staining. All metastatic foci in the lung were calculated microscopically to evaluate the development of pulmonary metastasis.

For in vivo limiting dilution assay, HO8910 shTHOR cells and its control cells were mixed with Matrigel (BD) at a ratio of 1:1 and injected subcutaneously at various cell doses per mouse (*n* = 10). The tumor formation was evaluated at 8 weeks.

### Statistical analysis

GraphPad Prism (GraphPad Software, Inc., La Jolla, USA) was used for all statistical analyses. Statistical analysis was carried out using t tests or Bonferroni multiple comparison tests: *p < 0.05. A *p* value of less than 0.05 was considered statistically significant.

## Results

### THOR expression was elevated in human OC tissues and predicted the poor prognosis of OC patients

**Fig. 1:**
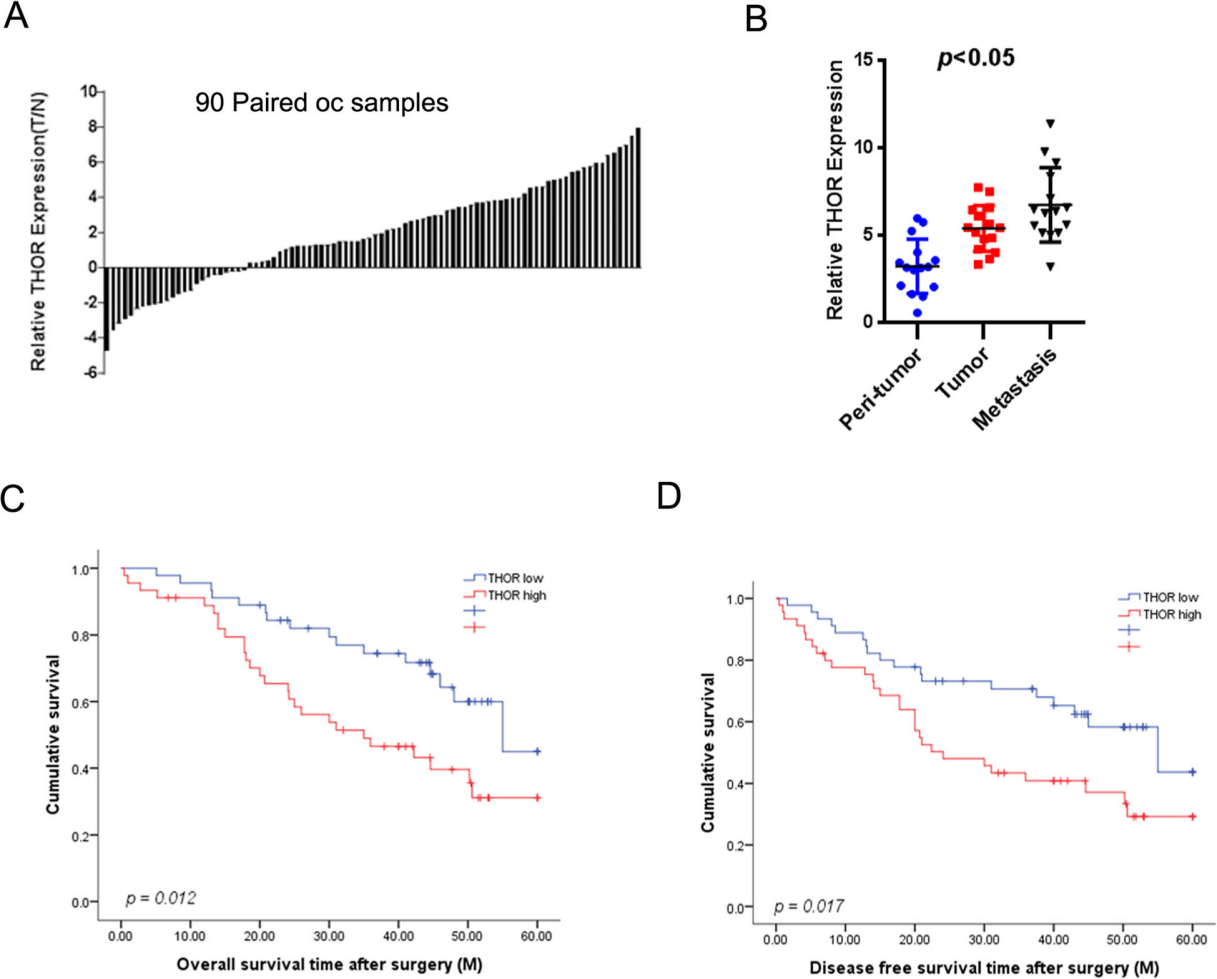
Upregulation of THOR in OC patient tissues. **a** The expression of THOR in 90 pairs of OC tumor (T) and normal tissues (N) was assessed by real-time PCR analysis, *p* < 0.05. **b** Comparison of THOR transcripts in paired peri-tumor normal tissues, OC tissues, and metastatic lesions using real-time PCR (*n* = 15), *p* < 0.05. **c** and **d** The total of 90 OC patients were divided into low THOR group (*n* = 45) and high THOR group (*n* = 45), and the recurrence and overall survival of the patients in the two groups were compared, *p* < 0.05

To explore the function of THOR in OC progression, the expression of THOR was checked in a large number of human OC tissues. As shown in Fig. 1a, THOR expression was dramatically upregulated in OC tumor tissues compared with the paired non-tumorous tissues. OC patients have high rates of metastasis, and the metastatic focus is a prognostic factor of poor prognosis in the patients [14]. As expected, THOR expression was increased in metastatic foci compared with the primary OC tissues, indicating THOR has important role in OC metastasis (Fig. 1b).

The cohort of 90 OC patients were equally divided into a “THOR high” group and a “THOR low” group based on the THOR expression. Univariate analysis demonstrated that the patients with low THOR levels possessed a lower risk of OC recurrence and a longer survival time after surgical resection than those with high levels (Fig. 1c and d).

### THOR promotes OC cells proliferation

**Fig. 2:**
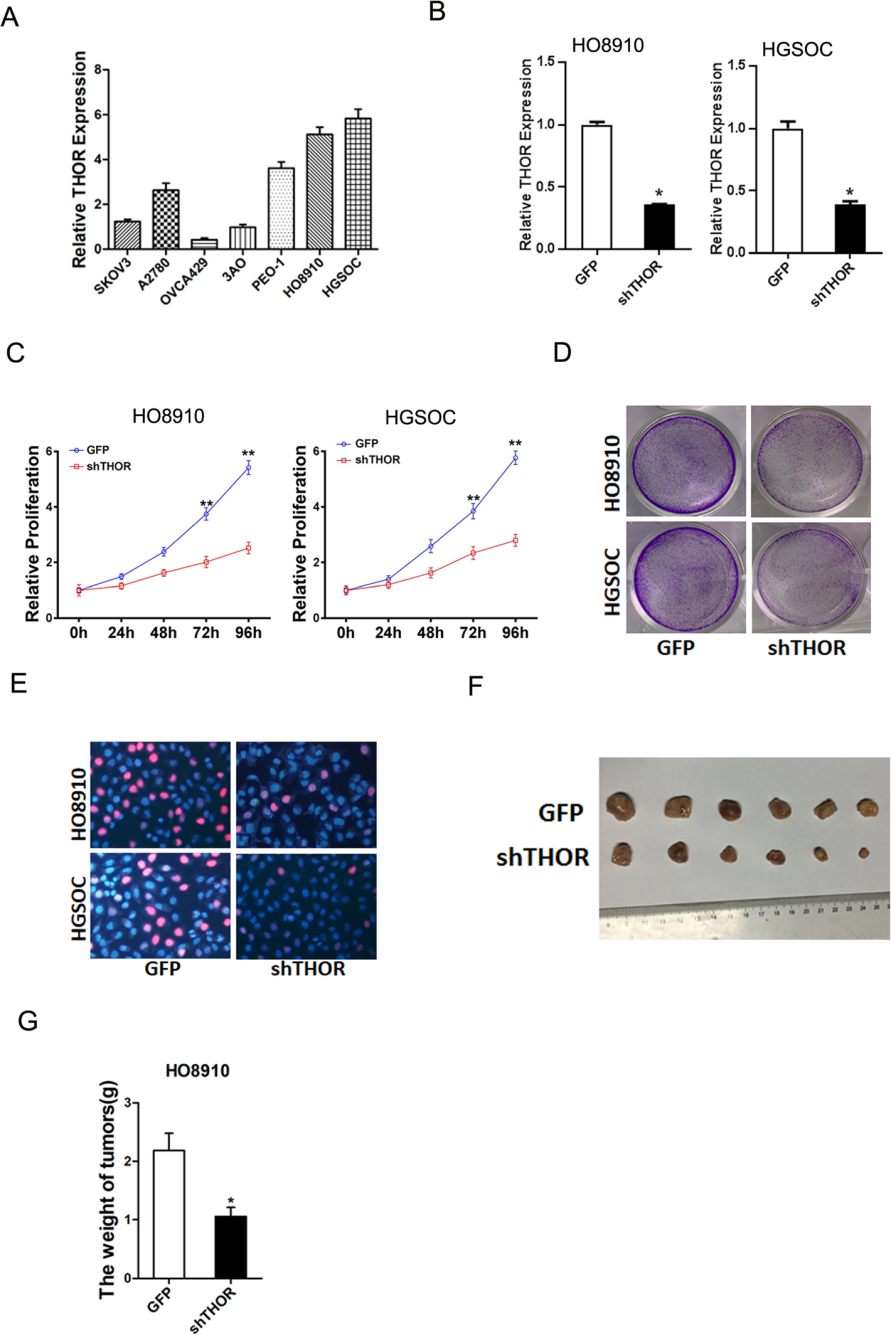
Knockdown THOR suppressed OC cells proliferation. **a** The expression of THOR in a great number of OC cells was checked by RT-PCR assay. **b** HO8910 and HGSOC cells were infected with shTHOR or its control lenti-virus, and then the cells were subjected to RT-PCR assays. **c** Cell proliferation of HO8910 shTHOR or HGSOC shTHOR cells and their control cells was measured by using CCK8 assays. **d** Colony formation assays of HO8910 shTHOR or HGSOC shTHOR cells and their control cells. **e** Cell proliferation was assessed using Edu immunofluorescence staining of HO8910 shTHOR or HGSOC shTHOR cells and their control cells. **f** HO8910 shTHOR and its control cells were subcutaneously injected into nude mice (*n* = 6) for xenograft assay. **g** The above tumor average weight in each group was shown, *p* < 0.05

To elucidate the role of THOR on OC cell behavior, we checked the expression of THOR in a great many OC cell lines and found THOR level in HO8910 and HGSOC cells was higher than other OC cell lines (Fig. 2a). So, we chose this two OC cell lines for further study. HO8910 and HGSOC cells were infected with shTHOR (5′-GGUGAACACAAUCGAGCAATT-3′) or control lenti-virus and the stable transfectants were obtained (Fig. 2b). As shown in Fig. 2c, THOR knockdown suppressed the proliferation of OC cells. In addition, HO8910 shTHOR and HGSOC cells shTHOR cells formed fewer and smaller colonies compared with their control cells (Fig. 2d). Furthermore, 5-ethynyl-2′-deoxyuridine (EdU) staining confirmed that THOR depletion inhibited OC cells growth (Fig. 2e). More importantly, THOR knockdown also inhibited the xenograft growth of OC cells in vivo (Fig. 2f and g). Taken together, the above results indicated that THOR promoted OC cell growth in vitro and in vivo.

### THOR drives OC cells metastasis

**Fig. 3:**
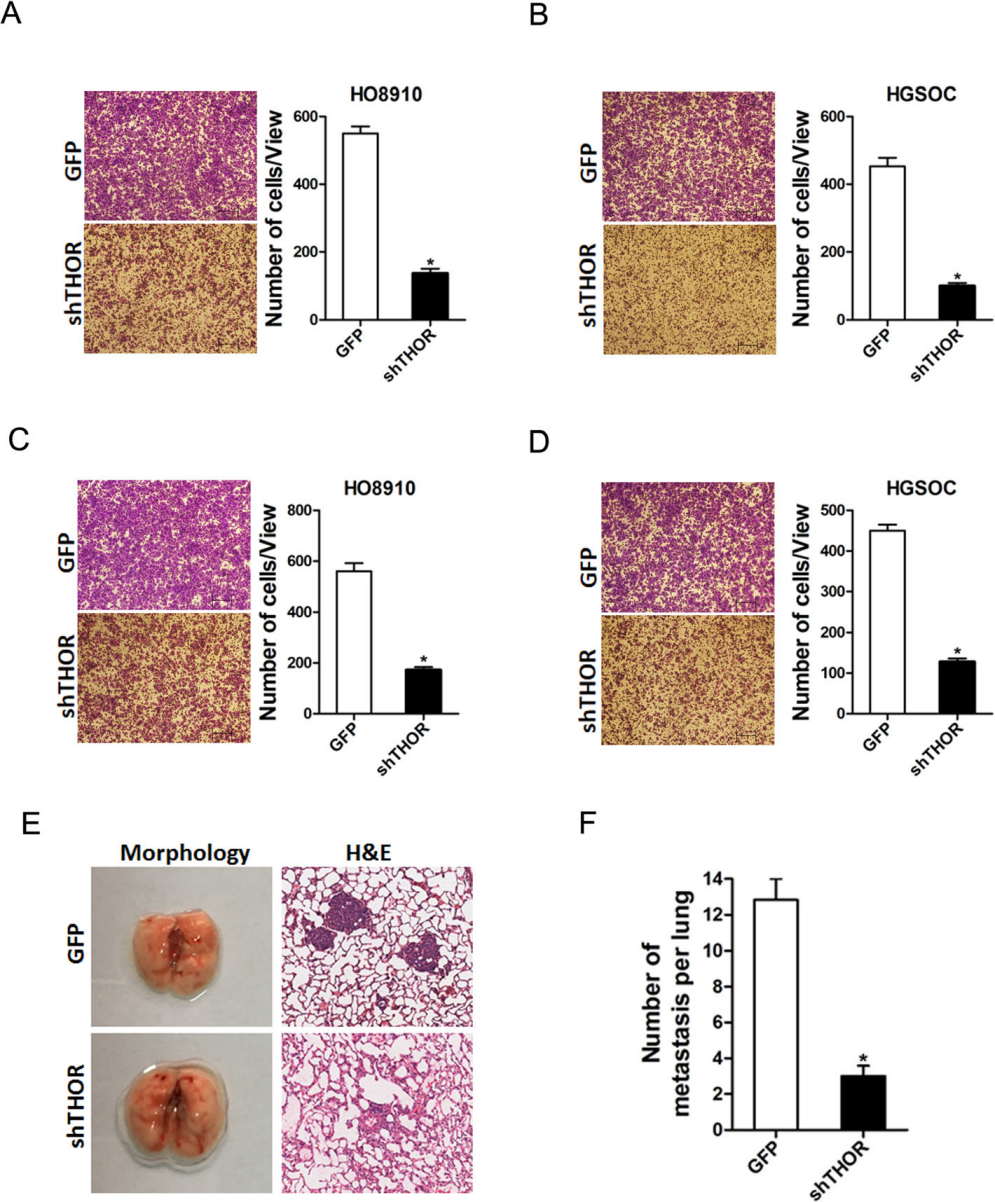
THOR depletion inhibited OC cells metastasis. **a** The migration of HO8910 shTHOR and its control cells was analyzed using polycarbonate membrane inserts in a 24-well plate, *p* < 0.05. **b** The migration of HGSOC shTHOR and its control cells was analyzed using polycarbonate membrane inserts in a 24-well plate, *p* < 0.05. **c** The invasive capacity of HO8910 shTHOR and its control cells was analyzed using Matrigel-coated Boyden chambers, *p* < 0.05. **d** The invasive ability of HGSOC shTHOR and its control cells was analyzed using Matrigel-coated Boyden chambers, *p* < 0.05. **e** Lung morphology and H&E staining of nude mice inoculated HO8910 shTHOR and its control cells via tail vein for 12 weeks. **f** The number of lung metastatic foci in each group (*n* = 7) were also calculated, *p* < 0.05

Next, we explored the role of THOR in OC cells metastasis; transwell assays and showed that migration ability was impaired in THOR-silenced OC cells (Fig. 3a and b). In addition, Matrigel invasion assays also revealed that THOR interference weakened the invasiveness of OC cells (Fig. 3c and d). Moreover, the in vivo lung metastasis experiment was performed. The results showed that no difference in lung morphology between shTHOR OC cells and control cells, while the H&E staining of nude mice showed much less lung metastasis foci in shTHOR OC cells (Fig. 3e and f). Collectively, our results showed that THOR promoted OC cell metastasis.

### THOR promotes ovarian CSCs expansion

**Fig. 4:**
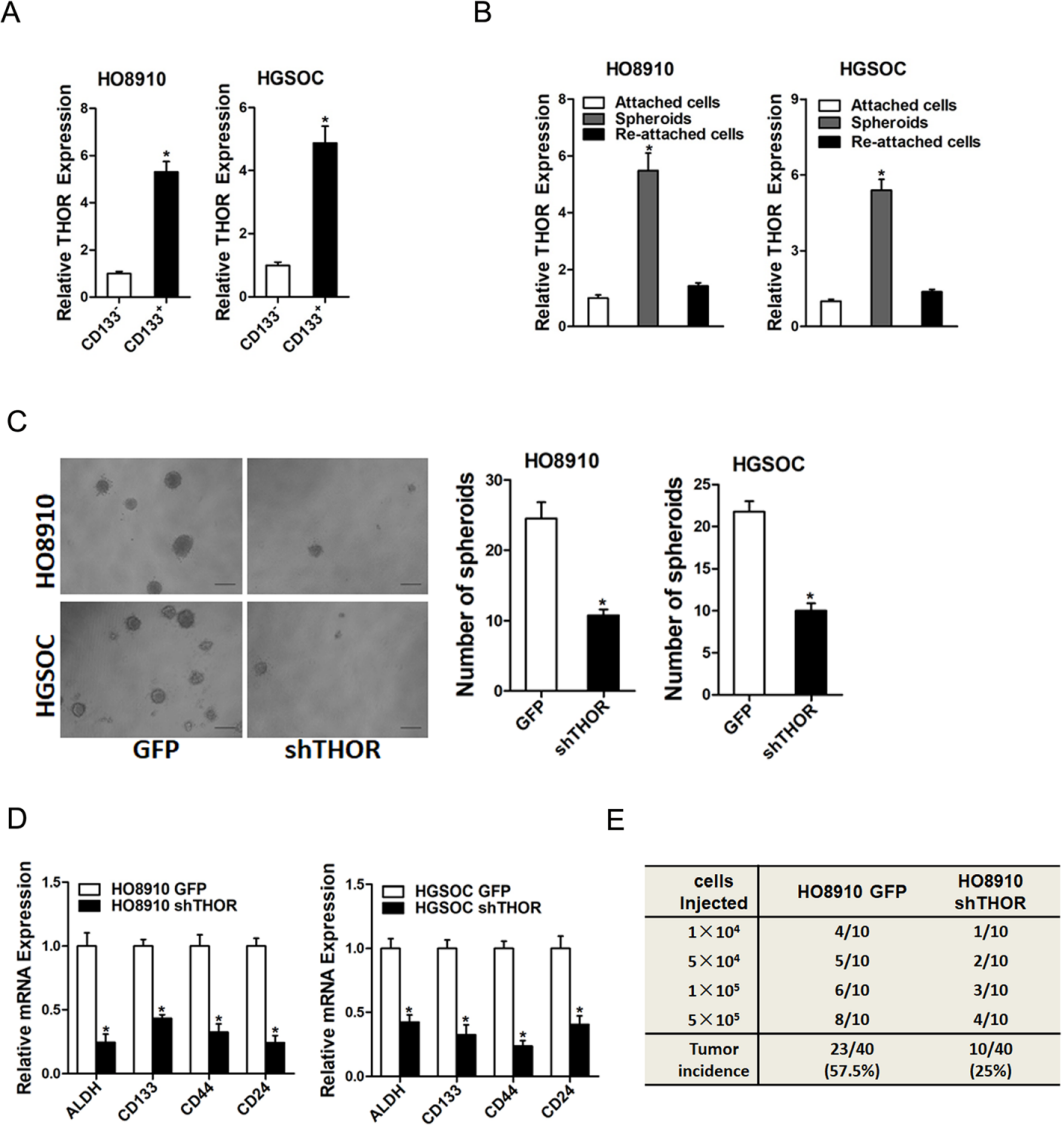
THOR promotes ovarian CSCs expansion. **a** RT- PCR analysis of THOR expression in flow cytometry sorted CD133^+^ OC cells relative to CD133^−^ OC cells. **b** OC cell–derived spheroids were trypsinized and cultured in attachment conditions. THOR level in spheroids versus reattached cells was compared by RT- PCR assay. **c** Representative image of spheres formed from shTHOR and control OC cells. The number of the formed spheres was counted and compared. **d** The ovarian CSCs markers in shTHOR and control OC cells were determined by real-time PCR. **e** In vivo limiting dilution assay of shTHOR and control sphere-derived OC cells. (*n* = 10)

It was reported that CD133 was well-accepted ovarian cancer stem cells (CSCs) marker [15]. As shown in Fig. 4a, THOR expression was dramatically increased in CD133-positive ovarian CSCs. Consistently, THOR expression was also upregulated in the self-renewing spheroids compared with the attached cells. Intriguingly, THOR level could be partially recovered when the spheroids cells reseeded in attached plates (Fig. 4b). Then we used THOR stably interference OC cells to explore the potential role in ovarian CSCs. As expected, THOR knockdown OC cells formed fewer spheroids compared with its control cells (Fig. 4c). Moreover, the expression of ovarian CSC markers was also suppressed in THOR interference spheroids (Fig. 4d). To further determine the effect of THOR on the tumorigenicity of ovarian CSCs, sphere-derived shTHOR or its control cells were inoculated into NOD-SCID mice. In vivo limiting dilution assay revealed that suppression of THOR significantly reduced tumor incidence (Fig. 4e).

### THOR promotes OC cell progression through the STAT3 signaling

**Fig. 5:**
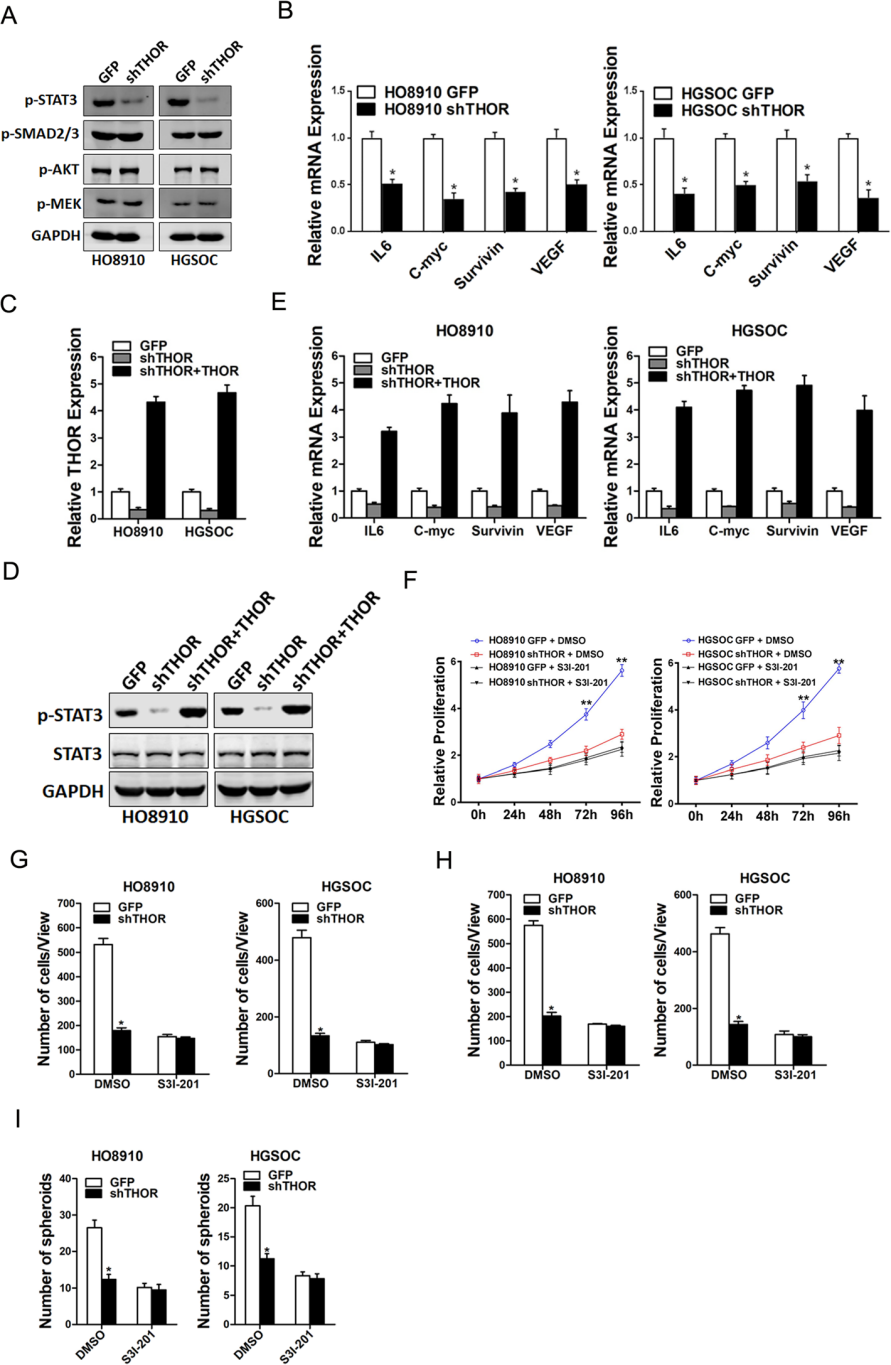
THOR promotes OC cells progression via STAT3 pathway. **a** The phosphorylation status of STAT3, SMAD3, AKT, and MEK in HO8910 shTHOR or HGSOC shTHOR cells and their control cells was determined by Western blot assays. **b** The mRNA expression of IL-6, C-myc, Survive and VEGF in HO8910 shTHOR or HGSOC shTHOR cells and their control cells was checked by real-time PCR. **c** The shTHOR OC cells were infected with THOR overexpression virus, and the overexpression effect was checked by RT-PCR assay. **d** The shTHOR OC cells were infected with THOR overexpression virus, and then subjected to western blot assay. **e** The shTHOR OC cells were infected with THOR overexpression virus, and then subjected to RT-PCR assay. **f** The proliferation of HO8910 shTHOR or HGSOC shTHOR cells and their control cells in the presence of S3I-201 (100 nM) or not was measured using CCK8 assays. **g** Migration assays were performed using OC shTHOR cells and its control cells in the presence of S3I-201 (100 nM) or not. **h** Invasion assays were performed using OC shTHOR cells and its control cells in the presence of S3I-201 (100 nM) or not. **i** Spheroid formation assays were performed using OC shTHOR cells and its control cells in the presence of S3I-201 (100 nM) or not

Numerous studies shown that TGF-β/SMAD, PI3-K/Akt, MAPKs and JAK/STAT3 signaling pathways have pivotal roles in various cellular processes, including cell growth, metastasis and apoptosis [16–19]. Herein our data showed that TGF-β/SMAD, PI3-K/Akt or MAPKs pathway was not influenced by THOR interference. However, STAT3 phosphorylation was inactivated by THOR knockdown in HO8910 and HGSOC cells (Fig. 5a). Moreover, the STAT3 downstream molecular factor was impaired in THOR knockdown OC cells (Fig. 5b). Next, we infected shTHOR OC cells with THOR overexpression virus (Fig. 5c). The results showed that the exogenous overexpression of THOR could rescue the STAT3 phosphorylation and other downstream effectors expression in THOR-knockdown ovarian cancer cells (Fig. 5d and e).

Then the special STAT3 inhibitor S3I-201 was used to confirm the role of STAT3 in THOR promoting OC cells growth, metastasis and self-renew. As expected, the inhibitor S3I-201 dramatically abolished the growth difference between *THOR* knockdown OC cells and control cells (Fig. 5f). Consistently, the inhibitor S3I-201 also eliminated the discrepancy in metastasis between THOR-silenced OC cells and their control cells (Fig. 5g and h). In addition, the inhibitor S3I-201 also abrogated the discrepancy of self-renewal ability between THOR-silenced OC cells and their control cells (Fig. 5i), suggesting that *THOR* promoted OC cell progression by activating STAT3 signaling.

### THOR promotes OC cells progression via IL-6 signaling

**Fig. 6:**
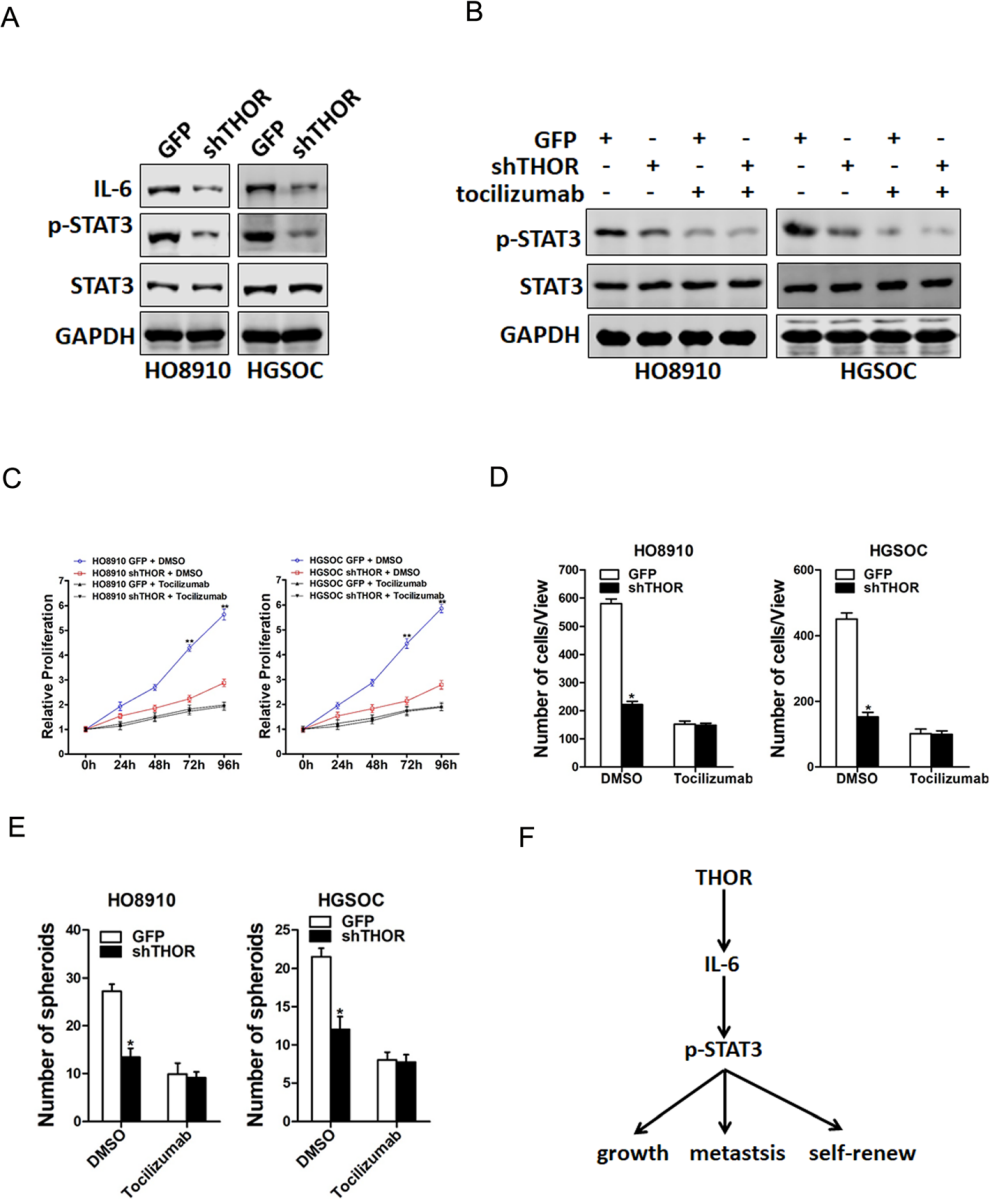
THOR promotes IL-6 activation in OC cells. **a** The protein expression of IL-6 in shTHOR OC cells and their control cells was determined by Western bolt assay. **b** shTHOR OC cells and their control cells were treated with tocilizumab (20 μg/mL) or DMSO, respectively, and then subjected to western blot assay. **c** The proliferation of shTHOR OC cells and their control cells in the presence of tocilizumab (20 μg/mL) or DMSO, respectively, was measured using the CCK8 assay. **d** Invasion assay was performed using shTHOR OC cells and their control cells with or without tocilizumab (20 μg/mL) treatment. **e** shTHOR OC cells and their control cells were treated with tocilizumab (20 μg/mL) or DMSO, respectively, and then subjected to spheroids formation assay. **f** Schematic diagram of the THOR/IL-6/STAT3 regulatory pathway in OC cell lines

Numerous studies showed that the activation of STAT3 is predominantly regulated by the upstream interleukin 6 (IL-6) families [20]. Our data showed that IL-6 protein level was dramatically downregulated in both HO8910 and HGSOC shTHOR cells (Fig. 6a). Furthermore, specific IL-6R inhibitor tocilizumab diminished the discrepancy of p-STAT3 between THOR knockdown OC cells and control cells (Fig. 6b). Moreover, specific IL-6R inhibitor tocilizumab dramatically abolished the discrepancy of proliferation, invasion and self-renewal between THOR knockdown OC cells and control cells (Fig. 6c-e). Taken together, our data showed that THOR promoted OC cells progression via activating IL-6/STAT3 signaling (Fig. 6f).

## Discussion

Ovarian cancer is one of in the female reproductive system and has a poor prognosis, which is related to its complex pathogenesis. The early symptoms of OC are not obvious and most patients are diagnosed at advanced stage [21]. The current treatments used for OC include some combination of surgery, radiation therapy, chemotherapy and targeted therapy [22]. While OC patients diagnosed at a late stage are usually incurable, and in that case the main goal of treatment is to improve the quality of life and prognosis [23]. In the present study, we demonstrated that THOR expression was upregulated in OC patients and might be a potential therapeutic target.

Emerging evidence indicts that lncRNAs are involved in embryonic development, stem cell self-renewal and differentiation, adipocyte differentiation and vascularization [24]. It is also accepted that lncRNAs participating in the initiation and progression of numerous human cancers and may be novel diagnosis markers and therapeutic targets [25]. For instance, lnc-DILC was reduced in liver cell stem cells and suppressed their expansion through inhibiting autocrine IL6/STAT3 pathway [26]. THOR is a newly discovered lncRNA, its function and mechanism of action in biological processes and diseases are not yet completely understood. It has been reported that THOR, a lncRNA with a cancer/testis expression pattern that exhibits a conserved interaction with IGF2BP1, potentially promoted oncogenesis. Moreover, ectopic expression of human THOR in zebrafish accelerated the initiation of melanoma. Latest studies also showed that THOR promoted hepatoma cells growth and metastasis via PTEN/AKT cascades [11]. However, the potential role of THOR in OC is unknown. In our study, we for first demonstrated that THOR was upregulated in OC tissues and predicted the poor prognosis of OC patients. Knockdown THOR inhibited OC cells proliferation, metastasis and self-renewal both in vivo and in vitro.

The existence of CSCs has been confirmed by numerous studies, and these cells have the ability to self-renew and the potential for generating heterogeneous malignant progenies [27, 28]. Most OC patients fail to eradicate tumors due to the existence of ovarian CSCs [29]. The understanding of the regulatory mechanisms for ovarian CSCs is currently limited. It was well-accepted that CD133 as the ovarian CSCs marker [30, 31]. In this study, our data showed that THOR level was upregulated in CD133^+^ ovarian CSCs. Spheroid culture of cancer cells is a routine approach to enrich CSCs. We also found that THOR expression was upregulated in OC spheroids. Moreover, we found that THOR knockdown inhibited the self-renewal ability and tumorigenesis capacity of ovarian CSCs.

STAT3 acts as a mediator of the signals initiated by the inflammation factor IL-6 cytokines, which regulate cell proliferation, differentiation and death [20, 32]. STAT3 expression has been reported to correlate with poor patient outcome in several types of cancer [33, 34]. In mice, STAT3 mutation has been linked to colorectal adenocarcinoma, increased systemic inflammation, and accelerated wound healing [35]. The altered activity of STAT3 was linked to chronic inflammation and somatic mutations that contribute to chronic colitis and the development of colorectal cancer [36]. In this study, we found that THOR played a positive role in OC cells and facilitated OC cell growth, metastasis and self-renew by activating STAT3 signaling. The STAT3 downstream molecular factor was also downregulated in THOR knockdown OC cells. It was well accepted that the activation of STAT3 is predominantly regulated by the upstream interleukin 6 (IL-6) families [37]. IL-6 families were playing important function in the regulation of initiation, progression and recurrence of human cancers. Then our results found that THOR upregulated p-STAT3 via IL-6. Specific STAT3 inhibitor S3I-201 or IL-6R inhibitor tocilizumab could also abrogate the discrepancy of proliferation, invasion and self-renewal between THOR knockdown OC cells and control cells.

We demonstrate for the first time that THOR expression is upregulated in OC tissues, and THOR shRNA silencing suppresses the growth, metastasis and self-renewal of OC cells. Moreover, THOR promotes OC cell progression by activating IL-6/STAT3 signaling. The findings of the present study not only shed new light on the mechanisms responsible for OC progression but also suggest that THOR might be a novel prognostic marker and a potential therapeutic target for OC.

## Data Availability

Data generated from the study are available from the corresponding author on reasonable request.
